# Minimum clinically important differences for the Functioning Assessment Short Test and a battery of neuropsychological tests in bipolar disorders: results from the FACE-BD cohort

**DOI:** 10.1017/S2045796020000566

**Published:** 2020-07-20

**Authors:** P. Roux, E. Brunet-Gouet, M. Ehrminger, B. Aouizerate, V. Aubin, J. M. Azorin, F. Bellivier, T. Bougerol, P. Courtet, C. Dubertret, J. P. Kahn, M. Leboyer, E. Olié, the FondaMental Advanced Centers of Expertise in Bipolar Disorders (FACE-BD) Collaborators, B. Etain, C. Passerieux

**Affiliations:** 1 Service Universitaire de Psychiatrie d'Adultes et d'Addictologie, Centre Hospitalier de Versailles, 177 rue de Versailles, 78157 Le Chesnay, France; 2 Université Paris–Saclay, Université Versailles Saint-Quentin-En-Yvelines, INSERM UMR1018, Centre de recherche en Épidémiologie et Santé des Populations, Equipe DevPsy, 94807 Villejuif, France; 3 Fondation Fondamental, Créteil, France; 4 Centre Expert Trouble Bipolaire, Pôle de Psychiatrie Générale et Universitaire (3/4/7), Hôpital Charles Perrens, Bordeaux, France; 5 Pôle de Psychiatrie, Centre Hospitalier Princesse Grace, BP489, 98012 Monaco, France; 6 AP-HM, Hôpital Sainte-Marguerite, Pôle de Psychiatrie, 13274 Marseille, France; 7 AP-HP Paris Nord, DMU Neurosciences, GHU Saint-Louis – Lariboisière – Fernand Widal, Département de Psychiatrie et de Médecine Addictologique, Paris, France; 8 Université de Paris, INSERM UMR-S 1144, Paris, France; 9 University Grenoble Alpes, CS 40700 38058 Grenoble cedex 9, Grenoble, France; 10 CHU de Grenoble et des Alpes, CS10217, F-38043 Grenoble, France; 11 Grenoble Institut des Neurosciences (GIN) Inserm U 836, Chemin Fortuné Ferrini, 38706 La Tronche, France; 12 Department of Emergency Psychiatry & Post-Acute Care, Academic Hospital of Montpellier, Montpellier University, Montpellier, France; 13 INSERM U1061, Montpellier, France; 14 AP-HP, Department of Psychiatry, Louis Mourier Hospital, Colombes, INSERM U894, Paris Diderot University, Sorbonne Paris Cité, Faculty of Medicine, France; 15 Fondation Santé des Etudiants de France (FSEF), Clinique Soins-Etudes, 51300 Vitry-le-François, France; 16 Université de Lorraine, Nancy, France; 17 AP-HP, Hôpitaux Universitaires Henri Mondor, DHU Pepsy, Pôle de Psychiatrie et d'Addictologie, Créteil 94000, France; 18 Université Paris Est, Faculté de Médecine, Créteil 94000, France; 19 Inserm, U955, Equipe Psychiatrie Translationnelle, Créteil 94000, France; 20 Department of Psychological Medicine, Centre for Affective Disorders, Institute of Psychiatry, Psychology & Neuroscience, King's College London, London, UK

**Keywords:** Attention, bipolar disorders, cognition, executive functions, memory, minimal clinical important differences, social functioning, speed processing, working memory

## Abstract

**Aims:**

Establishing the minimum clinically important difference (MCID) in functioning and cognition is essential to the interpretation of the research and clinical work conducted in bipolar disorders (BD). The present study aimed to estimate the MCID for the Functioning Assessment Short Test (FAST) and a battery of neuropsychological tests in BD.

**Methods:**

Anchor-based and distributive methods were used to estimate the MCID for the FAST and cognition using data from a large, multicentre, observational cohort of individuals with BD. The FAST and cognition were linked with the Clinical Global Impressions Scale-Severity (CGI-S) and Global Assessment of Functioning (GAF) using an equipercentile method. The magnitude of the standard error measurement (s.e.m.) provided another estimate of the MCID.

**Results:**

In total, 570 participants were followed for 2 years. Cross-sectional CGI-S and GAF scores were linked to a threshold ⩽7 on the FAST for functional remission. The MCID for the FAST equalled 8- or 9-points change from baseline using the CGI-S and GAF. One s.e.m. on the FAST corresponded to 7.6-points change from baseline. Cognitive variables insufficiently correlated with anchor variables (all *ρ* <0.3). One s.e.m. for cognitive variables corresponded to a range of 0.45 to 0.93-s.d. change from baseline.

**Conclusions:**

These findings support the value of the estimated MCID for the FAST and cognition and may be a useful tool to evaluate cognitive and functional remediation effects and improve patient functional outcomes in BD. The CGI-S and GAF were inappropriate anchors for cognition. Further studies may use performance-based measures of functioning instead.

## Introduction

Bipolar disorder (BD) is a complex and chronic illness characterised by lasting functional and cognitive deficits during all phases, including remission. Indeed, more than half the individuals with BD experience significant functional impairment in several domains, such as family and social life and work, outside the acute phases of the illness (Sanchez-Moreno *et al*., 2017). Some patients also present significant cognitive impairments even in the euthymic phase of the disorder (Roux *et al*., 2019). Traditionally, outcomes for patients with BD have been defined as the reduction of mood symptoms. However, the endpoints of randomised placebo-controlled trials (RCT) has recently shifted from clinical remission to functional recovery (Vieta and Torrent, 2016). In addition, as cognitive impairment is an important determinant of functional impairment in BD (Roux *et al*., 2017), functional recovery may be improved by cognitive remediation.

Recently, the number of clinical trials targeting cognition and psychosocial functioning in BD has markedly increased (Bellani *et al*., 2019), with RCTs showing promising results both for cognitive (Lewandowski *et al*., 2017) and functional (Torrent *et al*., 2013; Bonnin *et al*., 2016) remediation, while many trials are still ongoing (Strawbridge *et al*., 2016; Gomes *et al*., 2017; Ott *et al*., 2018). In such a context of the considerable deployment of resources, there is an urgent need to confirm whether statistically significant changes identified in clinical trials are beneficial to the individual in daily life. The smallest clinically meaningful improvement which can be perceived by patient caregivers is called the minimal clinically important difference (MCID). The MCID is crucial to accurately estimate the number of patients needed to treat in RCTs for continuous outcomes (Guyatt *et al*., 1998), such as cognition and functioning, by preventing the loss of power resulting from the dichotomisation of continuous scores when the MCID is unknown (Falissard *et al*., 2016). The MCID also plays a crucial role in interpreting cognitive and functional scale scores in a clinical setting. Until now, interpreting results from such instruments have relied on the personal experience of clinicians treating populations with BD and thus lack objectivity (Phillips *et al*., 2015). The MCID has been proposed as a more objective way to establish clinical relevance to changes in standardised instrument scores and can be used to assess the effectiveness of treatment.

Several methods used so far to estimate MCID have been classified according to whether they are anchor-based or distribution-based methods (Revicki *et al*., 2008). Anchor-based methods compare the instrument scores to an external gold-standard criterion, whereas distribution-based methods estimate the MCID based on a measure of the variability of the observed scores. We aimed to characterise the MCID for cognition and psychosocial functioning in BD using both anchor- and distribution-based methods, as combining the two strategies is widely recommended (Revicki *et al*., 2008). In this study, we investigated the MCID for psychosocial functioning for the Functioning Assessment Short Test (FAST), because this scale was specifically designed for BD, it is a domain-based measure of functioning (six domains: autonomy, occupational functioning, cognition, financial issues, interpersonal relationships and leisure (Rosa *et al*., 2007)), and it is a prevalent instrument in the literature (Chen *et al*., 2019). Cognition was investigated with a neuropsychological battery covering six relevant domains for BD. The use of multiple anchors is strongly recommended (Revicki *et al*., 2008). Thus, the two anchor dimensions selected in this study were global functioning and BD severity, which has been significantly associated with cognition in two meta-analyses (Bourne *et al*., 2013; Bora, 2018), as well as psychosocial functioning (Sanchez-Moreno *et al*., 2017).

## Methods

### Study design and characteristics of the recruiting network

This multicentre, longitudinal study included patients recruited into the FACE-BD (FondaMental Advanced Centers of Expertise for Bipolar Disorders) cohort within a French national network of 10 centres (Bordeaux, Colombes, Créteil, Grenoble, Marseille, Monaco, Montpellier, Nancy, Paris, and Versailles). This network was set up by the Fondation FondaMental (https://www.fondation-fondamental.org), which created an infrastructure and provided resources to follow clinical cohorts and comparative-effectiveness research in patients with BD. All procedures were approved by the local ethics committee (*Comité de Protection des Personnes Ile de France IX*) on January 18, 2010, under French law for non-interventional studies (observational studies without any risk, constraint, or supplementary or unusual procedure concerning diagnosis, treatment or monitoring). The board required that all patients be given an informational letter but waived the requirement for written informed consent. However, verbal consent was witnessed and formally recorded.

### Participants

The diagnosis of BD was based on the Structured Clinical Interview for DSM-IV-TR (SCID) criteria (First *et al*., 1997). Outpatients with type 1, type 2, or not-otherwise-specified BD, between 18 and 65 years of age, were eligible for this analysis. No criteria related to the current mood state at inclusion were used to preserve the variability of absolute and changed levels of functioning and cognition in this longitudinal observational cohort. However, individuals whose symptoms intensity was judged to be incompatible with the one-and-a-half-day evaluation at baseline were excluded (for instance, high suicidal risk, agitation, severe distractibility, disability to think or concentrate or severe indecisiveness).

### Assessment tools

The socio-demographic variables collected at inclusion were sex, age and education level.

#### Clinical assessments at inclusion and 12 and 24 months

The following clinical variables were recorded using the SCID: age at onset of BD, number and type of previous mood episodes, a subtype of BD and history of psychotic symptoms. Mania was measured using the Young Mania Rating Scale (YMRS; Young *et al*., 1978). Depression was measured using the Montgomery-Asberg Depression Rating Scale (MADRS; Montgomery and Asberg, 1979). We used a yes/no questionnaire for recording patient treatment at the three times of evaluation: lithium carbonate, anticonvulsants, antipsychotics, antidepressants or anxiolytics.

Domain-based psychosocial functioning was measured using the total score of the FAST, a short instrument comprising 24 items administered during an interview by a trained clinician. Two external criteria were used to anchor and calibrate the FAST and cognition. The first was the Clinical Global Impression-Severity (CGI-S) scale, which assesses the severity of the disorder (Guy, 1976). This tool was selected as an anchor because it is a well-established rating used by practising clinicians and is widely used for this purpose in the field of MCID (Duru and Fantino, 2008; Hermes *et al*., 2012; Falissard *et al*., 2016). The CGI-S was preferred to the CGI-I to avoid any memory bias during the 2-year follow-up. For the CGI-S, the minimum clinically important difference has been defined as the minimal observable difference between two adjacent categories, which is 1. A difference of 2 was considered to be mild, 3 moderate, 4 marked, 5 severe or great (depending on the direction) and 6 extreme. The second anchor was the Global Assessment of Functioning (GAF; Jones *et al*., 1995), which measures global functioning. It was chosen because it is highly used in BD (Chen *et al*., 2019), particularly as a reference measure of functioning (Bonnin *et al*., 2018). For the GAF, the minimum clinically important absolute difference has been defined as the range of the score within one category, which is 10. An absolute difference of 20 was considered to be mild, 30 moderate, 40 marked, 50 severe or great (depending on the direction), and 60 extreme.

#### The battery of cognitive tests at inclusion and 24 months

Experienced neuropsychologists administered the tests in a fixed order that was the same for every centre. Testing lasted approximately 120 min, including 5-to-10-min breaks. The standardised test battery complied with the recommendations of the International Society for BD (Yatham *et al*., 2010). This evaluation was not performed at T12. It included 11 tests, amongst which five were subtests from the Wechsler Adult Intelligence Scale (WAIS) version III (Wechsler, 1997*a*) or version IV (Wechsler *et al*., 2008), as the French version of the WAIS-IV started to be used as it became available. The battery evaluated six domains:

- *Processing speed*: Digit symbol coding (WAIS-III) or coding (WAIS-IV), WAIS symbol search and TMT part A
- *Verbal memory*: California Verbal Learning Test (Delis, 2000) short and long delay free recall and total recognition
- *Attention*: Conners’ Continuous Performance Test II (detectability, (Conners and Staff, 2000)
- *Working memory*: WAIS digit span (total score) and spatial span (forward and backward scores) from the Wechsler Memory Scale version III (Wechsler, 1997*b*)
- *Executive functions*: colour/word condition of the Stroop test (Golden, 1978), semantic and phonemic verbal fluency (Lezak, 2004), and Trail-Making Test (TMT) part B (Reitan, 1958)
- *Verbal and perceptual reasoning*: WAIS vocabulary and matrices

Raw scores were transformed to demographically corrected standardised *z*-scores based on normative data (Golden, 1978; Conners and Staff, 2000; Poitrenaud *et al*., 2007; Godefroy, 2008). Higher scores reflected better performance.

### Statistical analyses

#### Anchor-based MCID estimation

The Spearman rank correlation coefficient was used to quantify the association between the clinical anchors (CGI-S and GAF) and the instrument being investigated (FAST or cognition). Linking analysis aims to find corresponding points on different (in length or content), but correlated, tests (Lim, 1993). It has been recommended that the clinical anchors and the instrument being examined have a correlation threshold ⩾|0.30| (Revicki *et al*., 2008; Cheung *et al*., 2014). Thus, linking analyses were performed only for variables that showed a correlation above this threshold. Among the several available linking techniques, equipercentile linking is particularly useful, as it allows a non-linear relationship, with a symmetric attribution of random error in measurement between the two tests, which is not true, for example, for linear regression (Kolen and Brennan, 2013). This technique sets the cumulative distribution functions of the two tests as equal and identifies the scores on each scale that have the same percentile ranks. The kernel method for equating tests was applied using the package kequate for R (Andersson *et al*., 2013).

In a cross-sectional analysis, the clinical anchor (CGI-S and GAF) scores were initially mapped to the FAST and cognition using equipercentile linking techniques for values at baseline and 12 and 24 months. The average linking values across all time points were also computed. Changes in CGI-S and GAF scores were then linked to corresponding changes in the FAST and cognition between baseline and 12 and 24 months. The average linking values for changes across all time points were also computed.

#### Distribution-based MCID estimation

The distribution-based method estimates the MCID by comparing the observed change in the FAST and cognition to the variability in these instruments calculated in this study as the standard error of measurement (s.e.m.), which is more concordant with a clinically meaningful change than other distributive methods (McHorney and Tarlov, 1995; Eisen *et al*., 2007). The formula for the s.e.m. is 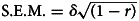, where *δ* is the standard deviation (s.d.) and *r* is the reliability as measured by the intraclass correlation coefficient. Previous studies have shown that values between 1 and 1.96 s.e.m. approximate the MCID (Wyrwich, 2004; Rejas *et al*., 2008; Falissard *et al*., 2016). To calculate the s.d. of the FAST and cognition, a subset of the population with stable symptomatology during the follow-up period was chosen by identifying individuals whose CGI-S score did not change from baseline to 24 months, a method similar to that used by several authors (Duru and Fantino, 2008; Hermes *et al*., 2012). The s.d. of the FAST and cognition scores for this population at baseline was used for the s.e.m. calculation. The intraclass correlation coefficient was calculated using a two-way mixed model of FAST and cognition at baseline and 24 months.

## Results

### Participants

The breakdown of participants at each time point was as follows: baseline, 1422; 1 year, 742 (47.8% of the participants were lost); and 2 years, 571 (59.8% of the participants were lost). Participants were included between January 2009 and October 2015. Their socio-demographic, clinical and functional characteristics at inclusion are presented in Table 1. A current mood episode was present in 15.5% of individuals at inclusion.

**Table 1.** Participants’ socio-demographical, clinical and functional characteristics at inclusion

Variable	Mean	s.d.	Range	Number of data points
Age (years)	41.1	11.6	18/65	1422
Educational level (years)	14.3	2.6	5/20	1380
Age at onset (years)	24.1	9.4	2/60	1363
Number of major depressive episodes	5.7	6.3	0/41	1161
Number of hypomanic episodes	3.5	6.3	0/41	1005
Number of manic episodes	1.2	2.4	0/32	1355
Number of mixed episodes	0.4	1.6	0/32	1204
MADRS (0–60)	9.3	8.4	0/42	1418
YMRS (0–60)	2.4	3.8	0/23	1419
CGI Severity (1–7)	4.1	1.5	1/7	1418
GAF (1–100)	65.9	13.6	20/100	1377
FAST total (0–72)	21.3	14.6	0/69	1402
		Percentage		
Sex (males)	39.2			1422
Diagnosis	48.9 (Type 1)	38.7 (Type 2)	12.5 (NOS)	1422
History of psychosis	42.8			1195
Current mood episode	12.3 Depressive	2.2 (Hypo)manic	1.1 Mixed	1324
Antidepressant	26.2			1230
Lithium Carbonate	24.5			1230
Anticonvulsant	35.2			1230
Antipsychotic	26.1			1230
Anxiolytic	22			1230

MADRS, Montgomery Åsberg Depression Rating Scale; YMRS, Young Mania Rating Scale; CGI, Clinical Global Impression scale; GAF, Global Assessment of Functioning scale; FAST, Functioning Assessment Short Test.

The number of participants who benefited from the neuropsychological evaluation was 1221 at inclusion (41% had WAIS-IV) and 366 at 2 years. The results of the neuropsychological tests are presented in Table 2.

**Table 2.** Participants’ neuropsychological performance at inclusion and the 2-year follow-up

Domain	Test	Variable	Inclusion				2 years			
			Mean	s.d.	Range	Number of data	Mean	s.d.	Range	Number of data
Speed of Processing	Digit/symbol coding		−0.4	1	−3/2.7	1205	0	0.9	−2.7/2.7	362
	Symbol search		0	1	−3/3	1200	0.3	1	−2.3/3	359
	TMT	Part A	0	0.9	−5.1/1.7	1210	0.3	0.8	−3.2/1.9	361
Verbal memory	CVLT	Short delay free recall	−0.2	1.1	−3.5/2.7	1195	0.1	1.1	−3.7/2.3	357
		Long delay free recall	−0.3	1.2	−4.5/2.5	1196	0.2	1.2	−4.2/2.2	357
		Total recognition	0	0.9	−2.6/0.7	1182	0.3	0.7	−2.6/0.7	354
Attention	CPT	Detectability	−0.1	1	−2.3/2.3	916	0	1.1	−2.3/2.3	310
Working memory	Digit span	Forward & backward	−0.2	0.9	−3/3	1196	0	0.9	−2.3/3	361
	Spatial span	Forward	−0.2	0.9	−3/2.7	999	0	0.8	−2/2.3	288
		Backward	−0.2	0.8	−3/2.3	999	−0.1	0.9	−3/2.7	288
Executive functions	TMT	Part B	−0.4	1.3	−10.1/1.8	1199	−0.1	1.1	−4.3/1.8	361
	Stroop test	Colour/word	−0.1	1	−3/3	1196	0.1	1	−3/2.8	356
	Verbal fluency	Phonemic	−0.1	1.1	−2.8/3.7	1184	0	1.1	−3/3.9	356
		Semantic	−0.4	1	−4.2/3.5	1184	−0.2	1	−3/4.4	356
Reasoning	Vocabulary		0.4	1	−3/3	1180	0.5	1	−1.7/3	329
	Matrices		−0.1	0.9	−3.3/2.3	1177	0.2	0.9	−3/2.3	337

TMT, Trail Making Test; CPT, Continuous Performance Test; CVLT, California Verbal Learning Test.

### Linking the FAST to the CGI-S and GAF

#### Linking of cross-sectional scores with the anchor-based MCID estimation

The correlations between the FAST total score, CGI-S and GAF are presented in online Supplementary Table 1. The observed correlations were statistically significant at all time points (*p*-value < 0.001) for the CGI-S and GAF, with all absolute values of Spearman's rank correlation coefficients >0.4, thus allowing anchor-based linking analysis.

The results of the linking between the FAST total scores and the CGI-S scores at each measurement wave are presented in online Supplementary Fig. 1. An average CGI-S ranking of 1-‘normal’ corresponded to a FAST score of 0, 2-‘borderline’ to 3, 3-‘mildly ill’ to 8, 4-‘moderately ill’ to 18, 5-‘markedly ill’ to 29, 6-‘severely ill’ to 40 and 7-‘extremely ill’ to 54.

The results of the linking between the FAST total scores and the GAF scores at each measurement wave are shown in online Supplementary Fig. 2. An average GAF ranking of 20 (some danger of hurting self or others) corresponded to a FAST score of 72, 30 (serious impairment in communication or judgment, or inability to function in almost all areas) to 63, 40 (major impairment in several areas, such as work or school, family relations, judgment, thinking or mood) to 50, 50 (any serious impairment in social, occupational, or school functioning) to 39, 60 (moderate difficulty in social, occupational, or school functioning) to 27, 70 (some difficulty in social, occupational or school functioning, but generally functioning pretty well, has some meaningful interpersonal relationships) to 16, 80 (no more than slight impairment in social, occupational or school functioning) to 7, 90 (good functioning in all areas, interested and involved in a wide range of activities; socially effective, generally satisfied with life, no more than everyday problems or concerns) to 2, and 100 (superior functioning in a wide range of activities, life's problems never seem to get out of hand, is sought out by others because of his or her many positive qualities) to 0.

#### Linking of change scores with the anchor-based MCID estimation

**Fig. 1.**
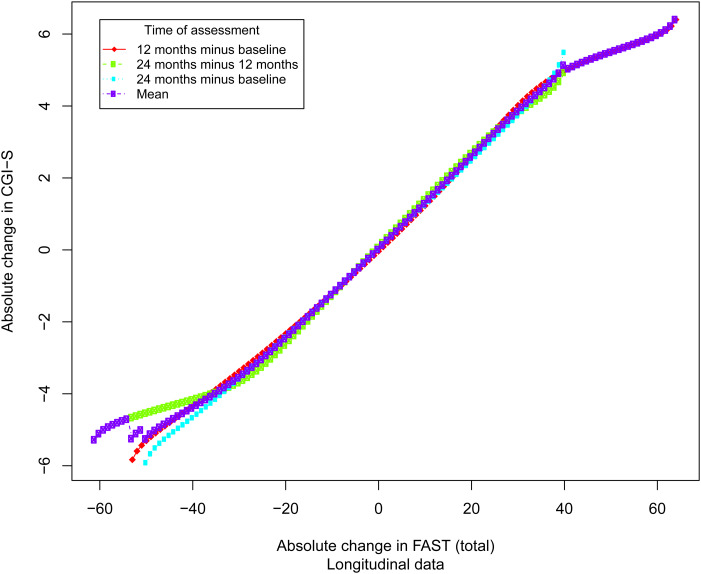
Equipercentile linking between changes in FAST and CGI-S.

**Fig. 2.**
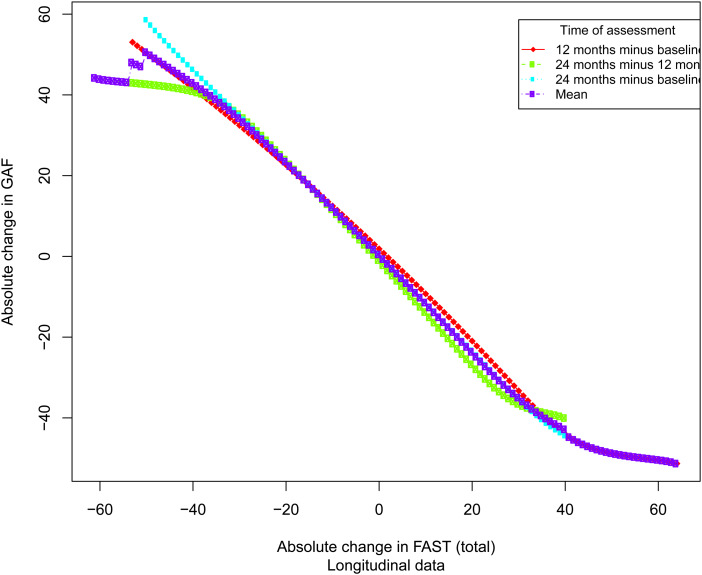
Equipercentile linking between changes in FAST and GAF.

Among participants, 35.6% and 42.2% presented no change in CGI-S and FAST, respectively. The number of occurrences in each level of change in CGI-S and GAF is reported in Table 3. The correlations between changes in the FAST, CGI-S and GAF are presented in online Supplementary Table 2. The observed correlations were statistically significant at all time points (*p* < 0.001) for the CGI-S and GAF, with all absolute values of Spearman rank correlation coefficients >0.3, thus allowing anchor-based linking analysis. The results of the linking between changes in the FAST total score and CGI-S at each measurement wave are shown in Fig. 1 and Table 3. The results of the linking between changes in the FAST total score and GAF at each measurement wave are shown in Fig. 2 and Table 3.

**Table 3.** Correspondence between different levels of change in the CGI-S, GAF and FAST

Level of change	Change in CGI-S	Number of data points for change in CGI-S	Correspondence between FAST and CGI-S changes	Change in GAF	Number of data points for change in GAF	Correspondence between FAST and GAF changes
Extreme worsening	6	0	⩾61		0	
Severe worsening	5	3	(41, 61)	⩽−50	1	⩾57
Marked worsening	4	18	(31, 41)	(−50, −40)	4	(36, 57)
Moderate worsening	3	53	(23, 31)	(−40, −30)	26	(25, 36)
Mild worsening	2	112	(16, 23)	(−30, −20)	174	(17, 25)
Minimum clinically important worsening	1	190	(8, 16)	(−20, −10)	179	(9, 17)
No change	0	604	(−8, 8)	(−10, 10)	737	(−8, 9)
Minimum clinically important improvement	−1	349	(−16, −8)	(9, 19)	367	(−16, −7)
Mild improvement	−2	208	(−25, −16)	(19, 29)	177	(−25, −16)
Moderate improvement	−3	126	(−35, −25)	(29, 39)	55	(−35, −25)
Marked improvement	−4	29	(−59, −35)	(39, 49)	22	(−48, −35)
Great improvement	−5	7	⩽−59	⩾50	5	⩽−49

CGI, Clinical Global Impression scale; GAF, Global Assessment of Functioning scale; FAST, Functioning Assessment Short Test.

The MCID for the FAST was equal to 8 or 9 points using the CGI-S and GAF (lower bound of the minimum clinically important improvement for CGI-S: 8; lower bound of the minimum clinically important worsening for CGI-S: 8; lower bound of the minimum clinically important improvement for GAF: 8; lower bound of the minimum clinically important worsening for GAF: 9). A change in FAST of 16 points was considered to be mild, 23 moderate and 31 marked.

### Distribution-based MCID estimation for the FAST

The sub-population for which there was no change in the CGI-S between inclusion and 12 months consisted of 233 individuals with a mean FAST score of 18.6 (s.d. = 26.3) at inclusion. The reliability of the FAST calculated as the intra-class correlation between the FAST scores at baseline and those at 12 months was 0.73, which computes to 1 s.e.m. = 7.6 and 1.96 s.e.m. = 14.9 FAST points.

### Linking cognition to the CGI-S and GAF

#### Linking of cross-sectional scores with the anchor-based MCID estimation

The correlations between cognition, CGI-S and GAF are presented in online Supplementary Table 3. The Spearman rank correlation coefficients were all ⩽0.2: it was thus not possible to perform an anchor-based analysis linking cognition with the CGI-S and GAF. However, certain observed correlations were statistically significant. The strongest negative association between the CGI-S and cognition was found for CPT-Detectability at 24 months (*ρ* = −0.15, uncorrected *p*-value = 0.007). The strongest positive association between the GAF and cognition was found for Verbal Fluency-Semantic at 24 months (*ρ* = 0.2, uncorrected *p*-value ⩽0.001).

#### Linking of change scores with the anchor-based MCID estimation

The correlations between changes in cognition, CGI-S and GAF are presented in online Supplementary Table 4. The absolute values of Spearman rank correlation coefficients were all ⩽0.2: it was thus not possible to perform an anchor-based analysis linking cognition with the CGI-S and GAF. A few observed correlations were statistically significant. The strongest negative association between changes in CGI-S and cognition was found for Digit/symbol coding (*ρ* = −0.18, uncorrected *p*-value = 0.001) and the strongest positive association between changes in GAF and cognition was found for Digit Span Forward & backward (*ρ* = 0.13, uncorrected *p*-value = 0.019).

### Distribution-based MCID estimation for cognition

The results are presented in Table 4. The MCID for cognition ranged from 0.45 (for Digit/symbol coding) to 0.93 (for TMT part B) for 1 s.e.m. and from 0.88 to 1.82 for 1.96 s.e.m.

**Table 4.** Distribution-based estimates for the minimal clinically important differences for cognition

Variable	N	ICC	1 s.e.m.	1.96 s.e.m.
Digit/symbol coding	113	0.78	0.45	0.89
Symbol search	112	0.69	0.56	1.1
TMT part A	115	0.61	0.59	1.15
CVLT short delay free recall	111	0.61	0.72	1.4
CVLT long delay free recall	111	0.71	0.67	1.31
CVLT total recognition	110	0.36	0.69	1.35
CPTdetectability	77	0.47	0.72	1.42
Digit span forward & backward	111	0.74	0.45	0.88
Spatial span forward	79	0.37	0.69	1.35
Spatial span backward	79	0.38	0.67	1.31
TMT part B	114	0.53	0.93	1.82
Stroop colour/word	113	0.73	0.54	1.06
Verbal fluency phonemic	113	0.64	0.67	1.3
Verbal fluency semantic	113	0.62	0.62	1.22
Vocabulary	104	0.8	0.46	0.9
Matrices	104	0.69	0.51	1

ICC, Intra Class Correlation; TMT, Trail Making Test; CVLT, California Verbal Learning Test; CPT, Continuous Performance Test.

## Discussion

This study estimated the MCID for the FAST, a widely used measure of domain-based functioning in BD, along with a battery of cognitive tests.

### Main findings and comparison with other studies

This is the first study to report the MCID in psychosocial functioning and cognitive performance in BD. We found an estimate of 8 or 9 for the MCID in the FAST total score with the anchor-based approach, which corresponded to the threshold of 7.6 found with the 1-s.e.m. distribution-based approach. These results suggest that a change below 8 for the FAST total score would not be clinically significant at the individual level. Despite different conceptual underpinnings, the anchored- and distribution-based estimations of the MCID for the FAST were very close, thus providing additional evidence of the validity of these estimates. The 1.96 s.e.m. distribution-based approach gave a more conservative threshold of 14.9 for the FAST total score.

Moreover, using an anchor point of >80 for the GAF for functional remission (Bonnin *et al*., 2018), we obtained a cut-off of ⩽7 for the FAST. Considering the transition between borderline and mildly ill for the CGI-S as another anchor point for clinical remission, we obtained exactly the same threshold of <8 for the FAST. This threshold is lower than the cut-off of 11 previously estimated in a sample of 101 participants (Bonnin *et al*., 2018). This small gap between the two studies can be explained by the higher depressive and manic symptomatology in our sample than that in the other, in which participants were strictly euthymic. Indeed, functional remission is more difficult to attain in cases of more pronounced mood symptoms. Selecting only euthymic participants when studying functioning may be problematic for the generalisability of results, as it excludes participants with a mild form of chronic or highly recurrent depression, who may yet benefit from functional remediation. By contrast, the present study used open inclusion criteria, allowing for selection of what is likely a generalisable population of outpatients with BD.

For cognition, the threshold correlation of |0.3| with clinical severity or global functioning was obtained for none of the cognitive tests. A meta-analysis reported a mean Pearson correlation between neurocognitive ability and functioning of 0.27 (Depp *et al*., 2012). However, the correlations were lower for clinician ratings (such as for the three scales used in this study) than performance-based tasks and real-world milestones, such as employment. Performance-based tasks may thus be better candidates for anchoring cognition on functioning than clinician-rated scales such as GAF or CGI. Subtle cognitive impairments might also be detected with a self-reported scale assessing cognitive complaints, such as the ‘Cognitive complaints in Bipolar disorder Rating Assessment’ (COBRA). This scale may be more closely associated with functioning than objective neuropsychological performance. Anchor-based MCID in cognition measured with COBRA should thus be explored in further studies. In the present study, the MCID was evaluated using only distribution-based methods: 1 s.e.m. of the MCID ranged from 0.5 to 0.9 s.d. and 1.96 s.e.m. of the MCID ranged from 0.9 to 1.8 s.d.. Very few studies have explored the MCID in the context of a neuropsychological battery. An observational study reported a similar range of 0.5–0.9 s.d. for 1-s.e.m. of the MCID in cognition for mild cognitive impairment (Phillips *et al*., 2015). In this study, anchor-based MCID in cognition ranged from 0.3 to 0.9. Another study investigating reliable cognitive changes in schizophrenia reported even larger values, between 0.7 and 1.7 s.d. (Gray *et al*., 2014).

The MCID found for cognition in this study may seem to be very large to be considered as minimally detectable by patients and clinicians. Several factors may explain this large MCID in cognition. First, one might speculate that the 1.96 s.e.m. of the MCID in cognition may have overestimated the true MCID, as the 1.96 s.e.m. of the MCID in FAST was larger than the anchor-based MCID in our study. One previous study has indeed reported that even the 1 s.e.m. of the MCID in cognition was slightly larger than the anchor-based MCID (Cheung *et al*., 2014). Secondly, the neuropsychological performances were heterogeneous in our observational study, as the participants were not selected on their cognitive performance, as opposed to RCT's investigating cognitive remediation or enhancement. A significant heterogeneity implies high s.d. in cognitive performance, leading to a large s.e.m. and MCID. The MCID in cognition must thus be interpreted with caution, as the s.e.m. only reflects a change that cannot be attributed to measurement error alone. The fact that it may estimate the MCID is only theoretical (some authors consider, for example, the s.e.m. measures a minimal detectable difference rather than a minimal clinically important difference (De Vet *et al*., 2011)) and should be corroborated with clinical anchors. Here, the MCID was evaluated within an observational study. The MCID may differ depending on whether the data were gathered in an observational study or clinical trial (Revicki *et al*., 2008). RCTs may overestimate anchor-based MCID, as substantial differences in outcomes are expected on a carefully selected population (Falissard *et al*., 2016). By contrast, a distribution-based MCID would underestimate values due to the homogeneity of the selected population. Observational study-based MCIDs may conversely be more reliable as they are not affected by therapeutic interventions or eligibility criteria (Falissard *et al*., 2016).

### Limitations

This study had several limitations. The first was the long-time interval between the two waves for calculating the distribution-based MCID for the functioning (1 year) and cognition (2 years). This may have led to an overestimation of the s.e.m., increasing the probability of a change to occur during the follow-up period, especially since previous reports showed an improvement in psychosocial functioning and cognition in this cohort (Ehrminger *et al*., 2019). However, we believe that such an overestimation bias may have been controlled by the fact that the distribution-based estimates were computed on a sample of patients with stable functioning. The influence of mood symptoms (Bonnín *et al*., 2014), medication (Roux *et al*., 2019) and trauma (Jimenez *et al*., 2017) has not been assessed in this study and these are variables that could have influenced the patient outcomes. Another significant limitation was the lack of a psychometrically validated MCID for the two gold-standard anchor measures (CGI-S and GAF), which were determined based on the expertise of the authors and how the two scales were elaborated and clinically anchored. A final drawback was the loss of more than half of the patients to follow-up. No survey was proposed to the non-completers; it was thus impossible to investigate the reasons for such a high rate of attrition.

### Clinical implications

We estimated the MCID for the FAST with a large representative sample using various complementary analytical techniques. The results were consistent, giving an estimation of 8 points. This result may provide clinicians with a better understanding of a commonly used measure of functioning in BD in both research reports and clinical practice. In light of the recent developments of functional remediation in BD, it is crucial to know whether newer interventions are sufficient to achieve functional recovery and a clinically relevant change in functioning. The results presented here aid in the transposition of trial results into practice.

Our results were less clear for the determination of the MCID for cognition, as changes in cognitive performance did not consistently correlate with changes in clinical severity or functioning. Further studies should use performance-based tasks to evaluate functioning as clinical anchors for cognition in BD, using, for example, the Brief University of California, San Diego (UCSD) Performance-based Skills Assessment (Patterson *et al*., 2001). Despite this limitation, our results provide the first estimates for interpreting cognitive changes in BD at an individual level; these results would also help in estimating the required number to treat for RCTs in the field of cognitive remediation in BD.

## Supplementary material

For supplementary material accompanying this paper visit http://dx.doi.org/10.1017/S2045796020000566. click here to view supplementary material

## Availability of data and materials

Data will not be shared as participants did not give their consent for sharing these data.
