# Supplementary material for: Minimum clinically important differences for the Functioning Assessment Short Test and a battery of neuropsychological tests in bipolar disorders: results from the FACE-BD cohort
Source: Epidemiol Psychiatr Sci. 2020 Jul 20;29:e144. doi: 10.1017/S2045796020000566 (PMC7372163; doi:10.1017/S2045796020000566)
Supplement: Supplementary file 1 [file S2045796020000566sup001.zip › S2045796020000566sup003.rtf]

Supplementary Table 1. Spearman correlations between the FAST total score, CGI-S, and GAF.


 	CGI-S	GAF	
Assessment	Rho	p 	Rho	p 	
Baseline	0.45	< 0.001	-0.64	< 0.001	
12 months	0.47	< 0.001	-0.7	< 0.001	
24 months	0.53	< 0.001	-0.71	< 0.001	
FAST: Functioning Assessment Short Test, CGI-S: Clinical Global Impression - Severity, GAF: Global Assessment of Functioning 
