# Supplementary material for: Minimum clinically important differences for the Functioning Assessment Short Test and a battery of neuropsychological tests in bipolar disorders: results from the FACE-BD cohort
Source: Epidemiol Psychiatr Sci. 2020 Jul 20;29:e144. doi: 10.1017/S2045796020000566 (PMC7372163; doi:10.1017/S2045796020000566)
Supplement: Supplementary file 1 [file S2045796020000566sup001.zip › S2045796020000566sup004.rtf]

Supplementary Table 2. Spearman correlations between changes in the FAST, CGI-S and GAF: 

Variable	Assessments	Rho	p	
CGI-S	12 months minus baseline	0.4	< 0.001	
	24 months minus 12 months	0.32	< 0.001	
	24 months minus baseline	0.4	< 0.001	
GAF	12 months minus baseline	-0.5	< 0.001	
	24 months minus 12 months	-0.39	< 0.001	
	24 months minus baseline	-0.47	< 0.001	
CGI: Clinical Global Impression scale, GAF: Global Assessment of Functioning scale
