# Supplementary material for: Minimum clinically important differences for the Functioning Assessment Short Test and a battery of neuropsychological tests in bipolar disorders: results from the FACE-BD cohort
Source: Epidemiol Psychiatr Sci. 2020 Jul 20;29:e144. doi: 10.1017/S2045796020000566 (PMC7372163; doi:10.1017/S2045796020000566)
Supplement: Supplementary file 1 [file S2045796020000566sup001.zip › S2045796020000566sup005.rtf]

Supplementary Table 3. Spearman correlations between cognitive variables, CGI-S, and GAF. 

 	 	CGI Severity
	GAF
	
Cognitive variable	Assessment	Rho	p	Rho	p	
Digit/symbol coding	Baseline	-0.11	< 0.001	0.18	< 0.001	
	24 months	-0.13	0.013	0.16	0.003	
Symbol search	Baseline	-0.07	0.011	0.15	< 0.001	
	24 months	-0.06	0.293	0.09	0.092	
TMT part A	Baseline	-0.04	0.176	0.07	0.013	
	24 months	-0.01	0.802	0.07	0.162	
CVLT Short delay free recall	Baseline	0	0.944	0.07	0.012	
	24 months	0.12	0.03	-0.01	0.81	
CVLT Long delay free recall	Baseline	0.02	0.595	0.07	0.013	
	24 months	0.08	0.144	0.02	0.723	
CVLT Total recognition	Baseline	0.04	0.172	0.01	0.674	
	24 months	0.09	0.104	0	0.991	
CPT Detectability	Baseline	-0.01	0.762	0	0.921	
	24 months	-0.15	0.007	0.15	0.01	
Digit Span Forward & backward	Baseline	-0.11	< 0.001	0.12	< 0.001	
	24 months	-0.12	0.024	0.15	0.006	
Spatial span Forward	Baseline	-0.07	0.039	0.07	0.031	
	24 months	-0.02	0.765	0.05	0.444	
Spatial span Backward	Baseline	-0.04	0.214	0.08	0.011	
	24 months	-0.11	0.057	0.14	0.021	
TMT part B	Baseline	-0.04	0.132	0.09	0.001	
	24 months	0.01	0.913	0.06	0.298	
Stroop Colour/word	Baseline	-0.01	0.712	0.06	0.049	
	24 months	-0.09	0.081	0.1	0.077	
Verbal fluency Phonemic	Baseline	-0.07	0.013	0.13	< 0.001	
	24 months	-0.09	0.101	0.13	0.017	
Verbal fluency Semantic	Baseline	-0.1	0.001	0.12	< 0.001	
	24 months	-0.13	0.014	0.2	< 0.001	
Vocabulary	Baseline	0.01	0.774	0.04	0.196	
	24 months	0.02	0.768	0.06	0.269	
Matrices	Baseline	0.03	0.276	0.01	0.639	
	24 months	0.1	0.075	0	0.949	
CGI: Clinical Global Impression scale, GAF: Global Assessment of Functioning scale, TMT: Trail Making test, CVLT: California Verbal Learning Test, CPT: Continuous Performance Test
