# Supplementary material for: Minimum clinically important differences for the Functioning Assessment Short Test and a battery of neuropsychological tests in bipolar disorders: results from the FACE-BD cohort
Source: Epidemiol Psychiatr Sci. 2020 Jul 20;29:e144. doi: 10.1017/S2045796020000566 (PMC7372163; doi:10.1017/S2045796020000566)
Supplement: Supplementary file 1 [file S2045796020000566sup001.zip › S2045796020000566sup006.rtf]

Supplementary Table 4. Spearman correlations between changes in cognition, CGI-S, and GAF. 

Variable	Cognitive variable	Number of observations	Rho	pva	
CGI-S	Digit/symbol coding	319	-0.18	0.001	
	Symbol search	316	-0.08	0.159	
	TMT part A	324	-0.13	0.016	
	CVLT Short delay free recall	318	-0.04	0.437	
	CVLT Long delay free recall	319	-0.05	0.408	
	CVLT Total recognition	315	-0.06	0.298	
	CPT Detectability	239	-0.1	0.121	
	Digit Span Forward & backward	317	-0.07	0.237	
	Spatial span Forward	238	0	0.955	
	Spatial span Backward	238	-0.01	0.841	
	TMT part B	323	-0.04	0.499	
	Stroop Colour/word	318	-0.03	0.627	
	Verbal fluency Phonemic	318	0.02	0.738	
	Verbal fluency Semantic	318	-0.02	0.752	
	Vocabulary	290	-0.07	0.262	
	Matrices	295	-0.07	0.202	
GAF	Digit/symbol coding	306	0.12	0.035	
	Symbol search	303	0.07	0.228	
	TMT part A	312	0.12	0.038	
	CVLT Short delay free recall	307	0.03	0.563	
	CVLT Long delay free recall	308	0.05	0.356	
	CVLT Total recognition	304	0.04	0.488	
	CPT Detectability	232	0.04	0.519	
	Digit Span Forward & backward	305	0.13	0.019	
	Spatial span Forward	227	0.03	0.64	
	Spatial span Backward	227	0.04	0.593	
	TMT part B	311	0.01	0.904	
	Stroop Colour/word	306	0.05	0.393	
	Verbal fluency Phonemic	306	0.03	0.56	
	Verbal fluency Semantic	306	0.04	0.513	
	Vocabulary	279	0.07	0.267	
	Matrices	283	0.04	0.524	
CGI: Clinical Global Impression scale, GAF: Global Assessment of Functioning scale, TMT: Trail Making Test, CVLT: California Verbal Learning Test, CPT: Continuous Performance Test
