# Supplementary figures and images for: Minimum clinically important differences for the Functioning Assessment Short Test and a battery of neuropsychological tests in bipolar disorders: results from the FACE-BD cohort
Source: Epidemiol Psychiatr Sci. 2020 Jul 20;29:e144. doi: 10.1017/S2045796020000566 (PMC7372163; doi:10.1017/S2045796020000566)

## Equipercntile linking between FAST and CGI-S

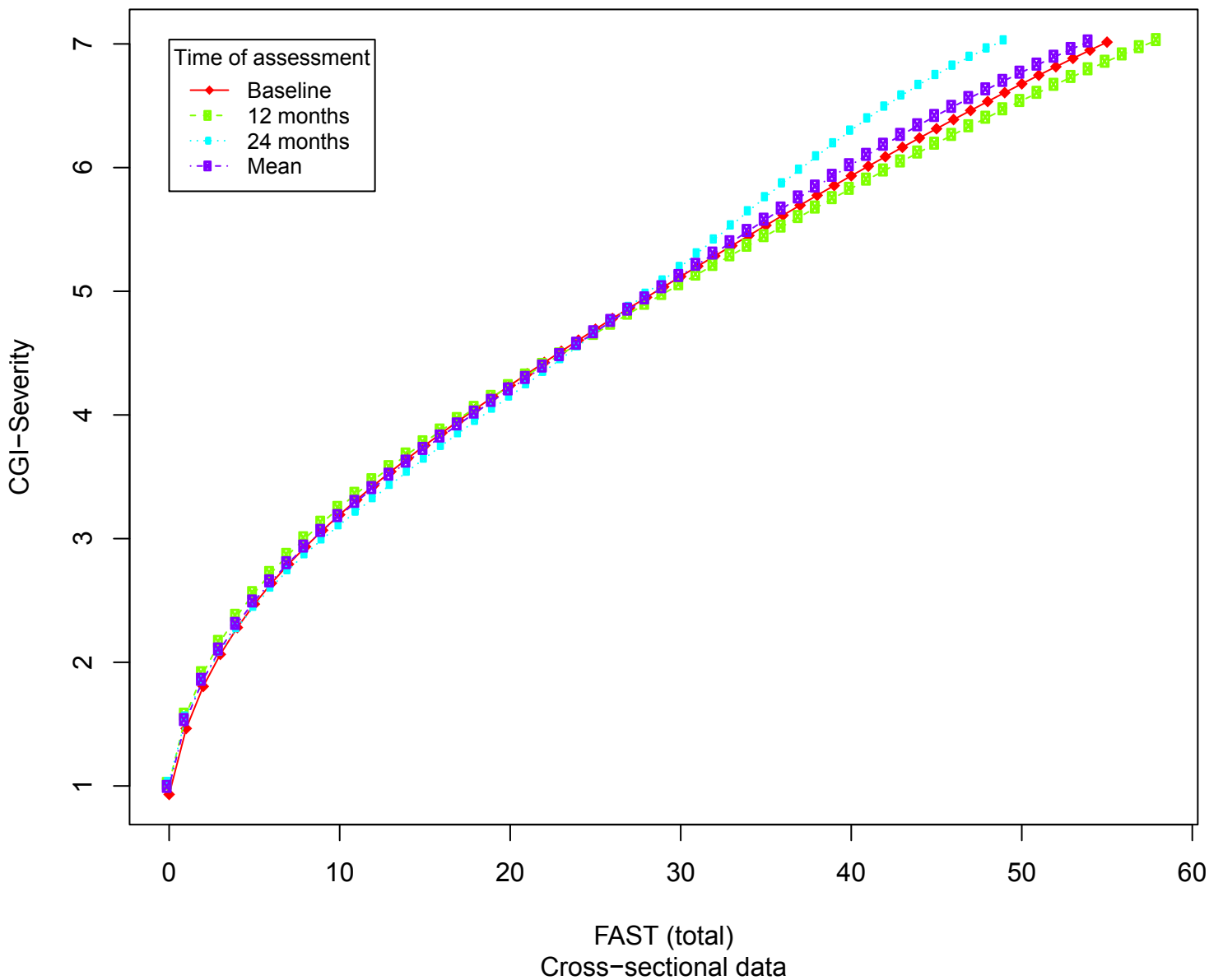

Supplement: Supplementary file 1 [file S2045796020000566sup001.zip › S2045796020000566sup001.pdf]

## Equipercntile linking between FAST and GAF

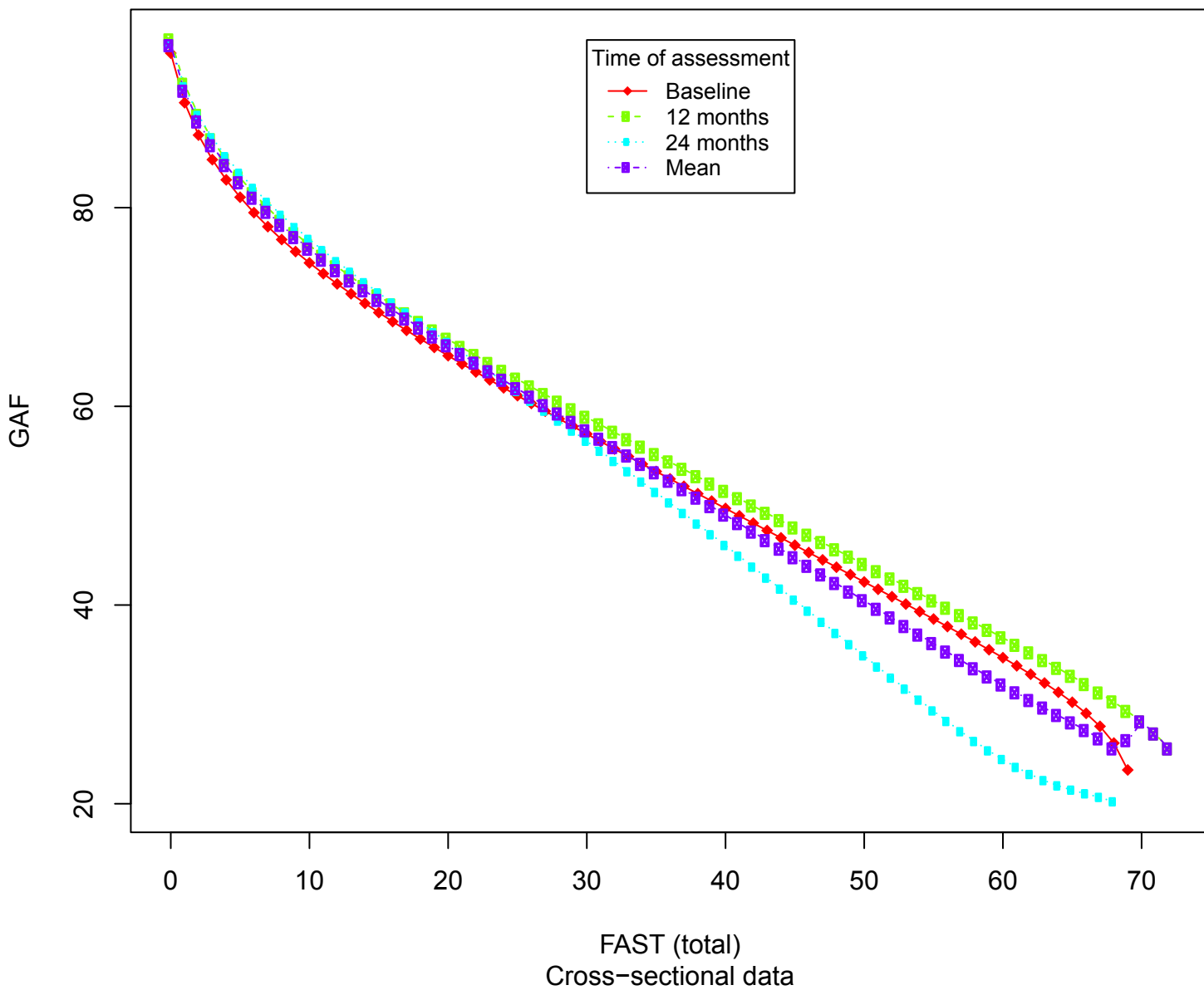

Supplement: Supplementary file 1 [file S2045796020000566sup001.zip › S2045796020000566sup002.pdf]
